# Appropriateness of elective caesarean deliveries in a perinatal network: a cross-sectional study

**DOI:** 10.1186/1471-2393-14-135

**Published:** 2014-04-09

**Authors:** Françoise Vendittelli, Marie-Caroline Tassié, Laurent Gerbaud, Didier Lémery

**Affiliations:** 1 The Clermont-Ferrand University Hospital, 58 Rue Montalembert, Clermont-Ferrand, 63003 Cedex 1, France; 2 Clermont Université, Université d’Auvergne, EA 4681, PEPRADE (Périnatalité, grossesse, Environnement, PRAtiques médicales et DEveloppement), 28 place Henri-Dunant, BP 38, 63001 Clermont-Ferrand, France; 3 AUDIPOG (Association des Utilisateurs de Dossiers informatisés en Pédiatrie, Obstétrique et Gynécologie), RTH Laennec Medical University, 7 rue Guillaume Paradin, 69372 Lyon Cedex 08, France; 4 Auvergne Perinatal Network, Clermont-Ferrand University Hospital, Site Estaing, 1 Place Lucie et Raymond Aubrac, 63003 Clermont-Ferrand, Cedex 1, France

**Keywords:** Audit, Birth, Caesarean, Delivery, Inappropriateness of care, Medical practice assessment

## Abstract

**Background:**

The overall caesarean rate in France has increased from 14.3% in 1994–1996 to 21.0% in 2010. This increased rate is a concern in all developed countries: delivery by caesarean induces both short- and long-term maternal complications, and its use requires careful reflection. The principal objective of this work was to describe the global appropriateness of indications for caesareans among a selected sample of planned caesareans performed within the Auvergne perinatal health network. The secondary objectives were to describe the inappropriate planned caesarean risk according to the maternity unit level and the impact of this medical assessment on the global caesarean rate in this network.

**Methods:**

This audit among maternity units belonging to the Auvergne perinatal network in France included women who had a planned caesarean at term, were nulliparous or primiparous, and had a singleton pregnancy in cephalic presentation or a twin pregnancy with twin 1 in cephalic presentation. We used the French guidelines issued from 1998 through 2010 as our benchmark for appropriateness.

**Result:**

We analysed 192 cases (100% of the records eligible for the audit). The rate of appropriate caesareans among these planned caesareans was 65.6%. Among the inappropriate caesareans, the rate of “maternal-preference” caesareans was 12.0% and the rate of “provider-preference” caesareans 22.4%. The risk of an inappropriate caesarean did not differ statistically between the level I and level II maternity wards, each compared to the level III hospital. The overall caesarean rate in our entire network decreased from 20.5% to 18.5% (p < 0.001) in the year after the audit. It also decreased in 8 of the network’s 10 maternity units, although the difference was statistically significant only in 2.

**Conclusions:**

About one third of planned caesareans were inappropriate in our sample and our audit appeared to have some effect on medical practice in the short run.

## Background

The overall caesarean rate in France increased from 14.3% in 1994–1996 to 17.2% in 2000–2002, 19.0% in 2006–2007 and 21.0% in 2010 [1,2]. This increase is a concern in all Western countries, including those in Europe [3,4]. Addressing planned caesareans in nulliparas is primordial because the risk of a repeat caesarean in another pregnancy is not only high but rising. For example, the rate of repeat caesareans in France has risen from 59.5% in 1994–1996 to 65.4% in 2006–2007 [1].

This continuous increase in the number of caesareans in France is a source of concern, as we see both from the French professional guidelines dealing directly or indirectly with indications for caesareans and from national reports [5-8]. Moreover, we know that the increase in the number of caesareans has not been accompanied by a proportional reduction in either neonatal complications or cerebral palsy [9,10] and that this mode of birth is associated with increased neonatal respiratory morbidity [11,12]. It also leads to both short-and long-term maternal complications, and its use requires careful reflection [13,14].

Variations in caesarean rates have been observed between countries, between regions, between hospitals in the same region and even between physicians in the same maternity ward [3,5,6,15]. The variation in this professional practice raises ethical questions and economic issues. It is therefore important to make tools available to maternity healthcare providers that allow them to analyse and thus improve their practices for planned caesareans.

The principal objective of this work was to audit and describe the global appropriateness of indications for caesareans among a selected sample of planned caesareans performed within the Auvergne perinatal health networks (RSPA) (part 1). The secondary objectives were to describe the risk of an inappropriate caesarean between the different level maternity wards (part 2), and the impact of this medical practice assessment on the global caesarean rate in the RSPA (part 3).

## Methods

### Materials

#### Description of the Auvergne perinatal health network (RSPA)

The RSPA comprises 14 healthcare facilities, of 4 types: one level III, 6 level II including one private maternity hospital, 3 level I and four local perinatal centres (where babies are not delivered); these account for 100% of the healthcare facilities managing pregnant women or newborns in the region of Auvergne. There are fewer than 14,000 deliveries each year in Auvergne. In 2009, the caesarean rate was 19.39% and in 2010, 20.18%. Table 1 describes the number of deliveries and the caesarean rate in each RSPA maternity ward in 2011 and 2012. Since 2008, the network has sent each maternity unit annual feedback on several indicators (rates of caesareans, episiotomies, etc.) together with the comparison of its own rates with those of the network.

**Table 1:** Number of deliveries, caesarean rate in Auvergne in 2011 and 2012, and number of selected cases by maternity unit

**Maternity unit**	Level*	**Date of file assessment**	**Number of analysed case files**	**Number of deliveries**		**Global caesarean rate (n)%**		
				**2011**	**2012**	**2011**	**2012**	**P value**
**1**	III	23/11/11	26	3440	3654	(n = 736) 21.4	(n = 717) 19.6	0.06
**2**	II	21/11/11	15	1281	1319	(n = 195) 15.2	(n = 175) 13.3	0.15
**3**	II	12/12/11	12	1233	1250	(n = 233) 18.9	(n = 228) 18.2	0.67
**4**	II	09/11/11	20	1150	1229	(n = 236) 20.5	(n = 223) 18.1	0.14
**5**	II	17/10/11	24	924	923	(n = 207) 22.4	(n = 191) 20.7	0.37
**6**	II	09/01/12	19	1190	1198	(n = 211) 17.7	(n = 225) 18.8	0.51
**7**	II	19/12/11	41	2385	2312	(n = 570) 23.9	(n = 463) 20.0	0.001
**8**	I	16/11/11	10	344	349	(n = 80) 23.3	(n = 70) 20.1	0.31
**9**	I	05/12/11	19	466	498	(n = 107) 23.0	(n = 78) 15.7	0.004
**10**	I	16/12/11	6	720	656	(n = 119) 16.5	(n = 109) 16.6	0.96
Total RSPA**	10 units	-	192	13,133	13,388	(n = 2,694) 20.5	(n = 2,479) 18.5	<0.001

*Level of hospital units. Level I maternity units provide prenatal care and delivery services for women with normal pregnancies with no particular risks. These units have no neonatology services. The woman will be referred to a more appropriate maternity ward in case of a maternal or fetal disorder. Level II maternity units have both an obstetrics unit and a neonatology nursery that provides 24/7 monitoring and special care for at-risk neonates, including those whose condition deteriorates after birth, regardless of whether they were born in the establishment. In particular, they are authorised to care for preterm infants born at or after 32 weeks gestation without any notable respiratory disease. Level III units also have a neonatal intensive care unit that allows them to provide 24/7 care for newborns in serious distress or at risk of death, again regardless of whether they were born in the establishment. In particular, they are authorised to care for infants born before 32 weeks and/or weighing less than 1500 g.

**RSPA=Auvergne perinatal network.

### Patients and participating centres

The population of our study was composed of women who had a planned caesarean (before or during labour) in a maternity unit in Auvergne and met these eligibility criteria: at or after 37 weeks of gestation, nulliparous or primiparous, and had a singleton pregnancy in cephalic presentation or a twin pregnancy with twin 1 in cephalic presentation. The exclusion criteria were: multiparity (>2), multiple pregnancies (≥3), dystocic or potentially dystocic presentations (breech and transverse), and emergency (unplanned) caesareans.

A planned caesarean was defined as a caesarean decided upon at least 48 hours before it was performed. A caesarean was defined as appropriate when it met at least one criterion for appropriateness as defined by national guidelines or if the experts conducting the review classified it as appropriate, even in the absence of a specific criterion. A “patient-preference” caesarean was defined as an inappropriate caesarean performed at the patient’s request, without any established medical or obstetric indication. A “provider-preference” caesarean was an inappropriate planned caesarean based only on the preference of one or several physicians practicing at that maternity ward.

### Design, interventions and data extraction

The principal endpoint of this cross-sectional study was the overall rate of appropriate caesareans in patients with a planned caesarean. The secondary endpoint was the global caesarean rate.

**Figure 1:**
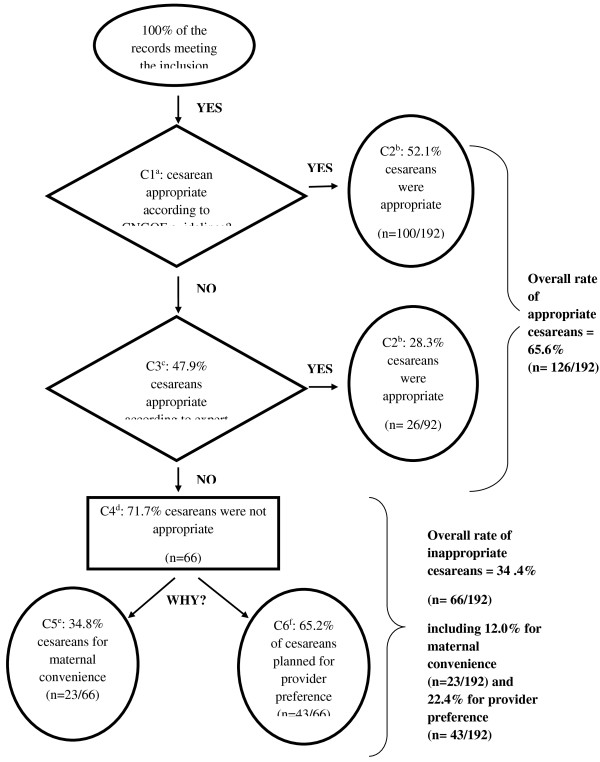
**Appropriateness of indication for caesarean: among planned caesareans in the Auvergne perinatal health network. **^a^Criterion n°1 (C1): The question is whether the caesarean is appropriate according to the French national references (French clinical practice guidelines, defined above). ^b^Criterion n°2 (C2): if the response to this question is yes, this caesarean is considered appropriate. ^c^Criterion n°3 (C3): if the response to this question is no, that is, that the published references allow this question to be answered (or not), then the experts present during this appropriateness review decide collegially whether the caesarean should be classified as appropriate or not. ^d^Criterion n°4 (C4): the caesarean is inappropriate according to the experts’ opinion. ^e^Criterion n°5 (C5): if the caesarean is inappropriate, we seek to determine if it was a maternal-preference caesarean, that it, performed only because the mother so requested. ^f^Criterion n°6 (C6): if the caesarean is inappropriate, we seek to determine if it was performed according to physician or department preference.

We chose as our method of analysis an audit, that is, a review of the appropriateness of care, a method that makes it possible to assess the adequacy of care in relation to the patient’s needs. This type of audit is based on a comparative approach that applies a set of predetermined, objective, standardised and validated criteria. If any single criterion was present, the care was considered appropriate. When no criterion in the list was present, we searched for the reasons that explained the inappropriateness of the care (Figure 1) [16]. This appropriateness review used as benchmarks the French national guidelines developed and published since 2000 by the relevant learned societies: the National Authority for Health (HAS) and the French National College of Gynaecologists and Obstetricians (CNGOF) [17-25]. There was no contradiction between any of the references, and a criterion of appropriateness had to appear at least once in a guideline to be defined as such.

This appropriateness review was based on the patient’s medical records (paper and/or computerised record and/or surgical reports, anaesthesia records, and correspondence).

The audit took place from October 2011 through January 2012 and covered all 10 RSPA maternity units. In each maternity ward, we studied the obstetric records of the planned caesareans performed from January 2011 through the moment of the study. All maternity units were asked to pull and provide, in chronological order, all medical records meeting our selection criteria. In each maternity ward, one or several senior gynaecologist-obstetricians participated in the appropriateness review (minimum 3 and maximum 7), together with the RSPA senior coordinator, a gynaecologist-obstetrician who served as the outside expert. The day of the audit, the unit’s delivery register was checked to verify that all records meeting our eligibility criteria were present for assessment. Data collection was totally anonymised: no record was made that identified either the patients or the professionals involved in the prenatal care or delivery. Similarly, each maternity ward was anonymised by the random allocation of numbers 1 through 10; they were, however, classified by level of care: I, II and III.

### Statistical analysis

The quality indicators reporting the appropriateness or inappropriateness of caesareans were presented as percentages. Continuous variables were compared by Student’s t test. Crude odds ratios (OR) of inappropriate caesareans were calculated according to maternity unit level, with their 95% confidence intervals (95%CI). Significance was set at 0.05. Data collection and analysis were performed with SAS software (version 9, SAS Institute, Cary, NC, 2002–2010).

## Results

### Number of files examined

Our study thus analysed 192 cases: 35 from the level I units, 131 from the level II facilities, and 26 from the level III reference hospital. These 192 dossiers included all of the records meeting our selection criteria during the study period, at the moment the audit took place in that maternity unit; both the study period and audit date necessarily differed for each maternity unit. Table 1 presents the number of cases analysed by establishment and the audit date in each.

### Description of the global appropriateness of the caesareans among this sample of planned caesareans in the RSPA (part 1)

The overall rate of appropriate caesareans among the planned caesareans was 65.6% (95%CI: 58.9-72.3). Of the 192 records examined, 52.1% (100/192) of the caesareans were appropriate, with at least one criterion of appropriateness present, and 13.5% (26/192) more appropriate based solely on professional judgement (Figure 1). The overall rate of inappropriate caesareans was therefore 34.4% (95%CI: 27.7-41.1). Among these planned caesareans, the overall rate due to maternal preference was 12.0% (95%CI: 7.4-16.6) and the overall rate due to provider preference 22.4% (95%CI: 16.5-28.3). The principal reasons for inappropriate provider-preference caesareans were: presumed foeto-pelvic disproportion (37.2%), especially after performance of X-ray pelvimetry (30.2%) and a previous caesarean (25.6%).

A greater proportion of appropriate than inappropriate planned caesareans was performed after 39 weeks (80.2% vs. 59.1%) (p = 0.002). Among the inappropriate planned caesareans, 43.5% of those in the maternal-preference group took place before 39 weeks and 39.5% of those in the provider-preference group (p = 0.76).

### Description of the appropriateness of planned caesareans by maternity unit level (part 2)

The risk of an inappropriate caesarean did not differ statistically between the level I and level II maternity wards, each compared to the level III hospital (Table 2). Nor did the reason for the inappropriate caesarean (physician preference vs. maternal request) differ between level I and level II, again each compared with the level III hospital (Table 3).

**Table 2:** Appropriate and inappropriate caesareans according to level of maternity units in Auvergne

**Level of maternities**	**Records (n = 192)**	**Appropriate caesareans (n = 126)**		**Inappropriate caesareans (n = 66)**		Crude OR*
		**Numbers (n)**	**% [95%CI]**	**Numbers (n)**	**% [95%CI]**	**[95%CI]**
**I**	35	21	60 [43.8-76.2]	14	40 [23.8-56.2]	2.80 [0.85-9.17]
**II**	131	84	64.1 [55.9-72.3]	47	35.9 [27.7-44.1]	2.35 [0.83-6.64]
**III**	26	21	80.8 [65.6-95.9]	5	19.2 [4.1-34.4]	1

*Inappropriate vs. appropriate caesareans.

**Table 3:** Causes of inappropriate caesareans according to maternity unit level within the Auvergne perinatal health network

**Level of maternity unit**	**Inappropriate caesareans (n = 66)**	**Maternal-preference caesareans (n = 23)**		**Physician-preference caesareans (n = 43)**		OR*
		**Numbers (n)**	**% [95%CI]**	**Numbers (n)**	**% [95%CI]**	**[95%CI]**
**I**	14	4	28.6 [4.9-52.2]	10	71.4 [47.8-95.1]	3.75 [0.44-31.62]
**II**	47	16	34.0 [20.5-47.6]	31	66.0 [52.4-79.5]	2.91 [0.44-19.20]
**III**	5	3	60 [17.1-100.0]	2	40 [0.0-82.9]	1

*Physician-preference vs. maternal-preference caesareans.

### Description of the short-term effect of our regional action (part 3)

We observed that between 2011 and 2012, the global caesarean rate in our perinatal network decreased from 20.5% to 18.5% (p < 0.001) (Table 1). The rate decreased significantly in the level II hospitals (20.2% to 18.3%; p = 0.002) and in the level I hospitals (20.0% to 17.1%; p = 0.04), but not in the level III hospital (21.4% to 19.6%; p = 0.06). When we compare the trends in caesarean rates in each unit, we observe rates fell for 8 of the 10 maternity units, although the difference was statistically significant for only 2 (one level II and one level I) (p < 0.05) (Table 1).

## Discussion

The rate of appropriate caesareans among these planned caesareans was 65.6%, in our audit. Among the inappropriate caesareans, the rate of maternal-preference caesareans was 34.8% and the rate of provider-preference caesareans 65.2%. Appropriateness of care is one of the most important issues of modern medicine [26]. As medical technologies become increasingly sophisticated and expensive, quality of care differs from quantity (and cost) of care. Appropriateness is a major topic that deals with physicians’ ability not only to provide good care but also to make the best choices. Unfortunately, appropriateness assessments are rarely performed for perinatal care [16]. Our strategy was based on the rating of appropriateness criteria; as proposed by Gertman and Restuccia [27], meeting a single appropriateness criterion sufficed to define care as appropriate. The criteria were based on a synthesis of French clinical practice guidelines from the relevant learned society (CNGOF) and from the HAS. These guidelines are based on different expert consensus procedures and are recognised as appropriate by French obstetricians. But because they do not cover all possible situations, in some cases we had to complete our assessment with professional expertise. Our method is not too hard to build, as the guidelines do not diverge much, but it requires medical expertise for the data collection. We had to select all the data necessary from a varied collection of medical records and we had to interpret the medical reasoning.

The use of expert guidelines may overestimate the real level of appropriateness, as physicians using a given medical technique tend to overrate its appropriateness [28]. This overestimation may be corrected by using a Rand Appropriateness Method (RAM), a process that brings expert and non-expert physicians together to rate appropriateness. The RAM, which is based on clinical scenarios, may also make it possible to avoid using medical expertise to rate appropriateness for clinical situations not planned by the medical guidelines [26,29]. It may also answer the question of necessity, if we analyse deliveries where a caesarean should have been performed [30]. But the RAM requires a much more laborious process for designing clinical scenarios and constructing rating grids. RAM would be very interesting for studying the appropriateness of emergency caesareans in France, a topic for which few national references exist.

Since this analysis was performed, the HAS published national guidelines for the indications for planned caesareans at term [31]. The consistency of our results with this new reference demonstrates that this work is both up-to-date and original. This appropriateness review could be used, in adapting the references, in other countries, such as England or in developing countries [4,32-34].

The study took place from 17 October, 2011, through 9 January, 2012, which explains in part the differences in the number of cases reviewed for each maternity unit (6–26). This number is correlated with the number of deliveries and with a planned caesarean policy that differed according to maternity unit. In an appropriateness review of planned caesareans, as in all analyses of practices, the “errors” found are often the same. It is therefore not necessary to have a large number of cases to be able to identify practices that deviate from those expected. In France, the perinatal health networks are considered an important lever for improving the quality and safety of patient care. In this study, the 100% participation rate of the maternity units in our network and the 100% assessment rate of all eligible caesareans in each maternity unit demonstrate the good regional validity of our results.

The planned caesarean rate is a good indicator of the quality of care in a maternity ward. Nonetheless, it is difficult to tell physicians what the correct rate of an intervention is, especially caesareans [35,36]. The mean rate is usually recommended, on the assumption that the highest and lowest rates are inappropriate, but this is not necessarily true. The ideal rate is that associated with the lowest level of maternal and neonatal morbidity and mortality, but it is difficult to ascertain in practice. The overall rate is thus actually a mean of different rates with a potentially wide range. It is simple to measure but does not allow easy comparisons of rates between different geographic locations or different maternity wards. It is more reliable, but more complicated, to work on standardised rates, whether they consider only women at low obstetrical risk or take obstetrical and medical risk factors into account (in multivariate analyses that make it possible to calculate an expected caesarean rate) [37,38]. These rates are essentially incomprehensible for physicians without substantial training and experience in statistics. A more qualitative analysis is more meaningful for physicians and optimises their awareness. The positive impact of such medical assessment should be translated by a decreased caesarean rate. This is the case in our study; the global caesarean rate decreased in the short term, in the first year after our study (p < 0.001). This decrease of the caesarean rate was noted in 8 over 10 of our maternity units but was statistically significant in only 2 of them.

We found a rate of inappropriate planned caesareans of 34.4% in our sample, but it is important to note that this is not the inappropriateness rate for all planned caesareans rate in Auvergne. If our denominator had been the number of planned caesareans, our inappropriateness rate would have been lower than that in our study (because formal indications for planned caesareans, including but not limited to preterm delivery for maternal or foetal reasons, or breech presentations at term were excluded from our audit). In Iran, a RAM was used to measure the appropriateness of caesarean deliveries [34,39]. Their caesarean rate was 36.6%, but the study also included several cases of unplanned caesareans (e.g., acute foetal distress and soft-tissue dystocia). In that study, the authors reported a rate of 47.2% for appropriate, 16.4% for “equivocal”, and 36.4% for inappropriate caesareans [39]. In our study, we sought to distinguish the inappropriate caesareans according to whether they were for the mother’s or the physician’s preference. We were surprised by the 13.0% rate for maternal-preference caesareans, which is close to the Iranian rate of 15.6% [39]. Data on this subject are sparse. One American study estimated this rate at 2.5% of births among a caesarean rate of 30% and in a context where guidelines on the subject exist [40,41]. An audit in Australia found a rate of 26.8% [42]. This maternal preference caesarean rate probably contributes to the observed increase in the overall caesarean rate, especially in countries with high caesarean rates and where patients’ decision-making autonomy has increased in recent years.

The factors found to be potential reasons for women’s requests in our study were essentially the same as in the literature [43]. The maternal-preference caesarean rate is often estimated in the literature indirectly from surveys of obstetric professionals. Accordingly, a survey of European practices among gynaecologist-obstetricians published in 2006 reported that acceptance of such caesareans was lowest in Spain (15.0%) and highest in the United Kingdom (79.0%) [44]. Different surveys have reported rates of obstetrician agreement to these requests of 19.0% in France [44] and 2.6% in Flanders [45]. It should be noted that the reported rate is not equal to the real rates observed and that the obstetrician can influence the mother’s preference [46]. These caesareans are considered unethical by the International Federation of Gynaecology and Obstetrics (FIGO) [47]. The HAS guidelines devote a section to planned caesareans on maternal request [31]. It specifies that this request “is not in itself an indication for a caesarean”. It is recommended that physicians seek to understand the specific reason for each request, discuss them as early as possible during the pregnancy, and report them in the medical file. Physicians can refuse to perform such caesareans. They must then refer the patient to a colleague [48]. Such a referral is feasible in large cities, but is considered difficult by professionals in the more remote maternity units in our region. There is often an emotional bond between the relatively few obstetricians in small cities or towns of Auvergne and their patients. The questions that must be asked are: is it possible to reduce the rate of caesareans for maternal requests? If so, how? There is however no evidence from randomised controlled trials upon which to base any practice recommendations regarding planned caesarean section for non-medical reasons at term [49]. In that case, if it is difficult to modify women’s behavior, might it be possible to modify that of professionals? The Cochrane meta-analysis analysed 16 studies [50,51]. Implementation of guidelines with mandatory second opinion could lead to a small reduction in caesarean section rates (adjusted risk difference = -1.9; 95%CI: -3.8 to -0.1). Peer review, including pre caesarean consultation, mandatory secondary opinions and post caesarean surveillance could lead to a reduction in repeat but not in total caesarean section rates. Guidelines disseminated with the endorsement of and support from local opinion leaders may increase the proportion of women with previous caesarean sections who are offered a trial of labour in certain settings (absolute difference 16.8%) and the number who had a vaginal birth (VBAC rates) (absolute difference 13.5%). Evidence that audit and feedback, training of public health nurses, insurance reform, external peer review and legislative changes could be effective was insufficient [51]. In a US setting, quite different from the French organisation of maternity units, a dedicated full-time labourist staff model has been associated with lower rates of caesarean delivery [52].

We have provided each maternity ward with its own rates of inappropriate caesareans and the reasons for their inappropriateness, compared with the means for the network. We have also organised a day of continuing medical education on caesarean deliveries in April 2012, in Auvergne. The short-term effect appears positive, and we can hope that this strategy will continue have a positive effect on practices.

## Conclusions

Our study revealed that the French healthcare providers do not always follow French guidelines about indications for caesareans. This conclusion had already been suggested by an earlier French study from the Audipog database about the impact of clinical practice guidelines in France [53]. Such an appropriateness review of caesareans, conducted within a perinatal health network, is feasible and would, more than a feedback of quantitative indicators to obstetricians, make it possible to quantify the rate of inappropriate caesareans, regardless of their cause. This type of medical qualitative assessment appeared to have some effect on medical practices in the short run, as this rate decreased in our perinatal network and in 8 of 10 maternity units, although the difference was significant in only two maternity units.

Other studies should be conducted among women to help understand and thereby reduce their preferences for caesareans, and a new assessment should then be conducted in our perinatal network.

## Competing interests

The authors declare that they have no competing interests.

## Authors’ contributions

FV designed the study, collected the data, and contributed to the analysis and interpretation of the data and to drafting the manuscript. MCT contributed to the conception of the study, data analysis, interpretation of data, and to drafting the manuscript. LG and DL contributed to the interpretation of data for the work and to the drafting of the manuscript. All the authors have read and approved the final manuscript.

## Authors’ information

FV, MD, PhD, is an associate professor in Public Health and an Obstetrician-Gynecologist.

MCT, Ms is a midwife.

LG, MD, PhD is a professor in Public Health.

DL, MD, PhD, is a professor in Obstetrics and Gynecology.

## Pre-publication history

The pre-publication history for this paper can be accessed here:

http://www.biomedcentral.com/1471-2393/14/135/prepub
